# Chitosan-Mediated Environment-Friendly Synthesis of Gold Nanoparticles with Enhanced Photonic Reactivity

**DOI:** 10.3390/nano12234186

**Published:** 2022-11-25

**Authors:** Ana Cazacu, Marius Dobromir, Ciprian Chiruță, Elena-Laura Ursu

**Affiliations:** 1 Department of Exact Sciences, “Ion Ionescu de la Brad” Iasi University of Life Sciences, 700490 Iasi, Romania; 2 Department of Exact and Natural Sciences, Institute of Interdisciplinary Research, “Alexandru Ioan Cuza” University of Iasi, 700506 Iasi, Romania; 3 Centre of Advanced Research in Bionanoconjugates and Biopolymers, “Petru Poni” Institute of Macromolecular Chemistry, 700487 Iasi, Romania

**Keywords:** chitosan-capped gold nanoparticles, colloidal system, photonic reactivity, surface plasmon resonance, biosensing

## Abstract

We developed a very simple, efficient and environment-friendly synthesis method for the manufacturing of high-performance chitosan-capped gold nanoparticles that could be used for biosensing applications. Gold nanoparticles were prepared through the spontaneous reduction of chloroauric acid by chitosan, which was used as both a reducing and a stabilizing agent. The samples were heated to a temperature of 60 °C under ultrasonic conditions. The composite system made of chitosan as a matrix and gold nanoparticles demonstrated a high stability in an aqueous buffer solution. The nanoparticles displayed an enhancement in photonic performance compared with the same property of individual components as a result of surface plasmon resonance at the interface between the structural phases of the hybrid structure. The enhanced photonic reactivity of the hybrid nanostructure may offer new insights for future possible biosensing applications.

## 1. Introduction

Gold nanoparticles (AuNPs) have received increasing interest from biomedical researchers because they have an inert nature, good biocompatibility and the possibility of biomolecule attachment via thiol–gold affinity interactions [1,2]. By precisely engineering them with different sizes, shapes and surface chemistries, AuNPs can be tailored to a variety of applications such as biosensors for biochemical assays [3], contrast agents for bioimaging [4,5,6] or drug and gene delivery vehicles [7,8,9]. For example, Rajamanikandan et al. reported the construction of a fast, ultrasensitive and highly specific colorimetric platform based on β-cyclodextrin-functionalized AuNPs for the detection of cysteine in human urine and blood serum samples [10]. Hu et al. demonstrated that cardiovascular optical coherence tomography could be used to detect individual cells suspended in biocompatible fluids by using gold nanoshells as intracellular contrast agents [11]. Huo et al. designed and fabricated a DNA-mediated self-assembled nanostructure. The uniform sunflower-like nanostructure exhibited strong NIR absorption, showed a sensitive photothermal response and allowed the control of the transfection efficiency down to a minimum level [7]. Most of these applications were based on the surface plasmon resonance (SPR) phenomenon of AuNPs. Hence, a broad UV-Vis absorption band with a maximum in the range of 520–600 nm appeared from the collective oscillations of the conduction electrons in the presence of visible light [12]. The SPR is highly sensitive to the surrounding environment (the refractive index and the presence of functional molecules around the nanoparticles) and it depends on the particle size and shape [13,14].

Concerning the synthesis of gold nanoparticles, many methods have been reported in the literature that often involve harsh chemical conditions of work and organic solvents that make AuNPs incompatible for in vivo applications; i.e., being toxic to living cells. Thus, it is of paramount importance to choose adequate reducing agents to diminish the toxicity of AuNPs. Although different reduction agents have been reported for the synthesis of AuNPs such as hydrazine [15], sodium citrate [16], sucrose-NaOH [17], phosphates [18] or ammonium hydroxide [19], polysaccharides (such as chitosan) are thought to be one of the most suitable reducing agents due to their good bioactivity and biocompatibility [20].

There are valuable studies that describe the fabrication of AuNPs and their functionalization with chitosan. Banihashem et al. obtained gold nanoparticles from a reduction with sodium citrate; afterwards, they coated them with a chitosan-grafted-poly(N-vinylcaprolactam) copolymer. Gold nanoparticles with particle sizes ranging from 10 to 100 nm were loaded with cisplatin to target MCF-7 breast cancer cells and normal human mammary fibroblasts (HMF3A) [21]. Other authors have addressed the fabrication of chitosan-capped gold nanoparticles (the mean particle size of AuNPs was about 10 nm) by a hydrothermal method for the detection of Ag^+^ ions [22]. An additional work explored alternative methods for gene transfers using AuNPs synthesized with sodium borohydride (as a reducing agent) or chitosan oligosaccharide (as a reducing and stabilizing agent) [23]. AuNPs with a mean diameter ranging from 3 to 15 nm have been conjugated with chitosan, acylated chitosan and chitosan oligosaccharide and have been used to evaluate the transfection efficiency of DNA into an HEK-293 cell culture. Gubitosa et al. reported the development of a self-assembled supramolecular system based on the use of green-synthetized AuNPs and chitosan [24]. AuNPs were also synthesized by using *Punica Granatum* juice and functionalized by chitosan wrapping for the adsorption of ellagic acid.

Nevertheless, most of these studies offer limited information about SPR characterization and the role of the chitosan molecular weight on the nanocomposite physicochemical reactivity.

As a consequence, we developed a facile and green method using a low quantity of gold for the production of gold nanoparticles in chitosan with an enhanced and controllable photonic activity as a function of the chitosan molecular weight and glycerol composition. In the samples that we prepared, chitosan acted as a matrix and was the surrounding medium in which the gold nanoparticles were formed. A systematic study of the obtained systems to evaluate their reactivity performance and their stability was performed. The average size of the AuNPs obtained by this method varied between 8 and 22 nm according to the transmission electron microscopy analysis.

## 2. Materials and Methods

### 2.1. Materials

Synthesis precursors were purchased from Sigma-Aldrich (Sigma-Aldrich, Saint Luis, MO, USA) and were used without further purification. These were hydrogen tetrachloroaurate (III) trihydrate (HAuCl_4_·3H_2_O), chitosan, acetic acid and glycerol. Different types of chitosan in terms of the molecular weight (Mw) and polydispersity index (PI) were used, including those with a practical grade (PG, Mw = 263.8 kDa, PI = 3.8), a medium molecular weight (MMW, Mw = 213.9 kDa, PI = 3.6) and a low molecular weight (LMW, Mw = 114.3 kDa, PI = 2.7). To prepare the precursor solutions, Milli-Q ultrapure water at a resistivity of 18.2 MΩ·cm was used.

### 2.2. AuNP Preparation

Polymer stock solutions were prepared by dissolving 0.1 g/L chitosan with different molecular weights in a solution of 1% acetic acid (*v*/*v*). The crystals of HAuCl_4_·3H_2_O were dissolved in Milli-Q ultrapure water to obtain a solution of HAuCl_4_ with a concentration of 1 mM. Samples of gold and chitosan were then obtained by mixing different ratios of chitosan and HAuCl_4_ solutions to obtain a final volume of 40 mL for each sample. The ratios were calculated in order to obtain gold concentrations of 0.06, 0.12, 0.18, 0.24 or 0.30 mM in the respective sample. AuNPs were formed by subjecting the samples for 10 min to an ultrasonic field with a frequency of 20 kHz and amplitude at the radiating surface of the probe of 80% using a Sonopuls HD 4100 Bandelin homogenizer (Bandelin Electronic GmbH & Co. KG, Berlin, Germany). As a consequence of this method, the solutions were both stirred and heated to a temperature of 60 °C.

The reaction mechanism involved in the formation of gold nanoparticles consists, firstly, of the metal salt anions (${\mathrm{AuCl}}_{4}^{-}$) binding by electrostatic attractive forces to the protonated amino groups (-NH_2_) at the C-2 position in the chitosan; secondly, of the simultaneous reduction in the Au^3+^ ions with the oxidation of the (-CH_2_OH) groups at the C-6 position and the (-CHO) groups at the C-1 position; and thirdly, of the agglomeration of the reduced Au atoms and the formation of gold nanoparticles that are further stabilized by the chitosan [25,26,27,28].

The names, gold concentration and pH of the samples are summarized in Table 1. The name was given in relation to the type of chitosan used and the final gold concentration in the solution.

For the photonic reactivity enhancement, a quantity of each AuNP solution was mixed with twice its volume of glycerol with different concentrations (0, 5, 15, 25, 50, 75 and 100% *v*/*v* in water). Thus, the final glycerol concentration from the samples varied from 0 to 66.67%. These solutions were analyzed by UV-Vis spectroscopy and dark-field microscopy.

### 2.3. Sample Characterization

XPS measurements were performed on a Physical Electronics PHI 5000 VersaProbe (Ulvac-PHI, Inc., Chigasaki, Japan) instrument equipped with a monochromated AlKα X-ray source (hν = 1486.6 eV). The take-off angle of the photoelectrons was 45°. All the XPS peak positions were calibrated with respect to the C 1s peak with a binding energy of 284.6 eV.

The Fourier transform infrared (FTIR) spectra were recorded between 4000 and 400 cm^−1^ with a Bruker Tensor 27 (Bruker, Karlsruhe, Germany) spectrophotometer.

The morphology of the AuNPs was investigated by transmission electron microscopy (TEM) with a Philips CM100 (Philips Electron Optics, Eindhoven, The Netherlands) microscope. For this, a drop of 3 μL of each sample was deposited on a formvar-coated copper grid. The size of the nanoparticles and their distribution were determined from the TEM micrographs using the NIS Elements Basic Research software (Nikon Europe B.V., Amstelveen, The Netherlands).

The size distribution of the AuNPs in the solution and their stability as a function of the average zeta potential were evaluated with a Malvern Nano ZS ZEN3500 Zetasizer (Malvern, UK), at room temperature.

The optical properties were studied in the solution by investigating the UV-Vis absorption spectra in the range of 400–700 nm using a Hitachi U-2001 spectrophotometer (Hitachi High-Tech Corporation, Tokyo, Japan).

The plasmon resonance effect was revealed using dark-field microscopy with a Nikon Ti Eclipse inverted optical microscope (Nikon Europe B.V., Amstelveen, The Netherlands).

## 3. Results and Discussion

The color of the final sample was determined by the size and geometrical shape of the AuNPs and also by the mass of the polymer covering the NPs [29].

**Figure 1:**
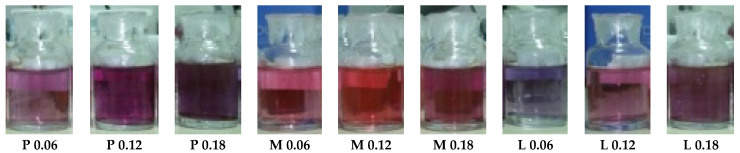
Different shades of AuNPs in solutions that remained stable for a long period of time (12 months).

As function of the amount of HAuCl_4_ solution and the type of the chitosan used, various shades of pink-violet were obtained, as shown in Figure 1, for the samples with gold concentrations of 0.06, 0.12 and 0.18 mM. The color was the first indicator to prove that AuNPs were being formed inside the respective polymer solution, which was demonstrated afterwards by different methods of investigation, as presented below.

**Figure 2:**
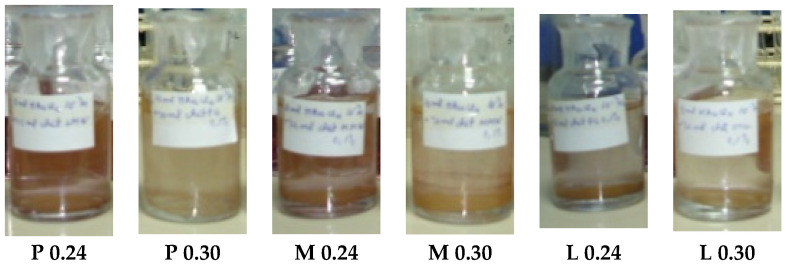
AuNP solutions with a concentration of 0.24 and 0.30 mM (unstable after a few weeks).

For all types of chitosan, an amount of 0.24 or 0.30 mM of the gold concentration in the initial solution determined the concentration gradients that, in time (a few weeks), led to the formation of Au microcrystallites seen as a brown precipitate. The solution with residual amounts of NPs was yellow-brownish (Figure 2). Although these samples initially had a pink color, they became unstable and precipitated due to the development of Au microcrystallites.

This proved that, for carefully chosen concentrations of gold precursor solutions and types of chitosan, the reaction chambers controlled the reaction–diffusion processes of nucleation and stabilized the NPs at a certain size.

XPS measurements were taken to determine the surface elemental composition of the samples and the chemical states of the gold (Au), oxygen (O), carbon (C) and nitrogen (N). Thus, the elemental composition data for the P 0.12 sample showed the following surface atomic concentrations of elements: 58.9% for C; 35.3% for O; 5.1% for N; and 0.7% for Au.

**Figure 3:**
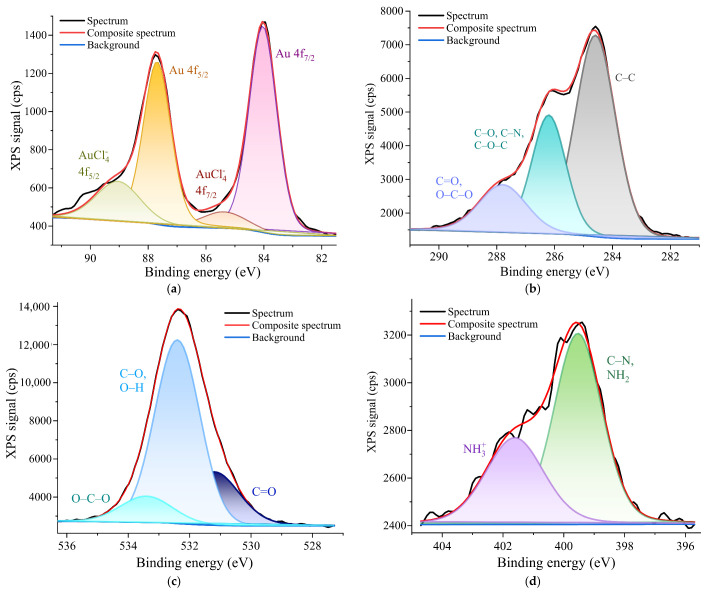
High-resolution deconvoluted XPS spectra of the P 0.12 sample for: (**a**) the Au 4f narrow scan peak; (**b**) the C 1s narrow scan peak; (**c**) the O 1s narrow scan peak; (**d**) the N 1s narrow scan peak.

The deconvolution of the Au 4f narrow scan peak of the P 0.12 sample (Figure 3a) showed a binding energy of the Au 4f_7/2_ peak at 84 eV and a higher binding energy at 87.7 eV of the Au 4f_5/2_ peak (with a separation of about 3.67 eV), which was specific to metallic Au^0^ [30]. The formation of Au^0^ was due to the high reducibility of the hydroxyl and amino groups on the chitosan surface [31]. The deconvolution also showed a peak at 85.5 eV (4f_7/2)_) and another one at 89.2 eV (4f_5/2)_), which were attributed to AuCl_4_^−^ [32]. These binding energy values were in good agreement with the values found in the literature.

The resolved C 1s spectrum revealed three peaks, as shown in Figure 3b. The C 1s peak at 284.6 eV was mainly assigned to the C-C chemical binding. The peak at 286.2 eV was assigned to C-O, C-N or C-O-C and the peak at 287.8 eV was assigned to C=O or O-C-O chemical bonds [33]. The O 1s spectra were deconvoluted into three components (Figure 3c): the first one at 531 eV corresponded with the C=O bond; the second one was at 532.2 eV, which is usually associated with C-O and O-H bonds; and the third one at 533.2 eV was assigned to the O-C-O chemical bond [34]. The N 1s XPS high-resolution spectra of the P 0.12 sample, shown in Figure 3d, showed evidence of two peaks at binding energies of 399.6 eV and 400.9 eV. The first peak was assigned to C-N/NH_2_ chemical bindings [35] or could be attributed to an NH_3_^+^-AuCl_4_^−^ interaction [36], demonstrating the successful bonding between the AuNPs and the chitosan matrix. The second peak was associated with the presence of two amino groups in an ammonium form (NH_3_^+^) [35].

**Figure 4:**
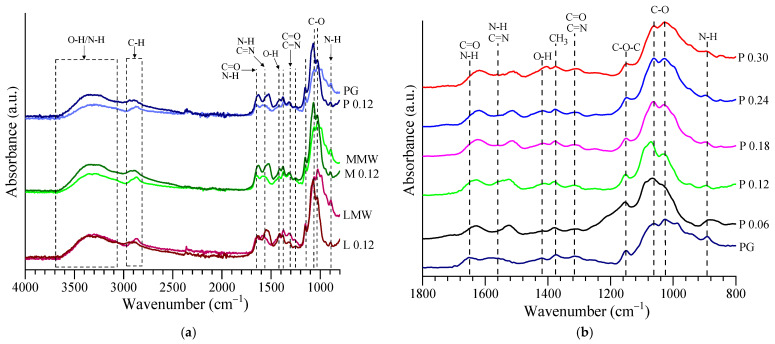
FTIR spectra of (**a**) used chitosan and lyophilized samples corresponding with a gold concentration of 0.12 mM and (**b**) all gold concentrations in the case of PG chitosan.

Figure 4 shows the FTIR spectra of the different types of chitosan and a few lyophilized samples of AuNPs. The characteristic spectrum of chitosan presents specific peaks for amide I at ~1653 cm^−1^ (C=O stretching, coupled with NH_3_^+^ asymmetric bending), amide II at ~1560 cm^−1^ (N-H and C=N in-plane bending), amide III at ~1312 cm^−1^ (C=O and C=N stretching) and O-H in-plane bending at 1419 cm^−1^. It also presents a broad band in the region of 3000–3670 cm^−1^ attributed to the coupled contribution of the O-H and N-H stretching vibrations given by the intramolecular and intermolecular hydrogen bonds, respectively. The bands at ~2915 cm^−1^ and ~2872 cm^−1^ were attributed to the C-H asymmetric and symmetric stretching vibrations, respectively; the peaks at ~1375 cm^−1^ and ~1154 cm^−1^ were attributed to CH_3_ symmetric bending and C-O-C asymmetric stretching, respectively. The C-O stretching vibrations peaks from ~1061 and ~1021 cm^−1^ and the N-H wag (for the primary and secondary amines) from ~893 cm^−1^ were specific for the polysaccharide structure of the chitosan. The characteristic bands/peaks of the chitosan FTIR spectrum found in this study were comparable with the values reported by other researchers [37,38,39,40,41,42].

An FTIR analysis was employed to attest the interactions between the chitosan and the AuNPs, knowing that, by mixing two or more components, the chemical interactions would lead to a change or shift in the characteristic peaks [43,44].

As can be seen from Figure 4a and Table 2, the differences in the spectra obtained from the chitosan powders and lyophilized AuNP samples for the broad band observed in the range of 3000–3670 cm^−1^ were due to residue water molecules that remained in the samples after lyophilization. The major changes in the characteristic bands from the fingerprint region (800–1700 cm^−1^) were due to the interactions between the functional groups of the chitosan and the atoms of gold. An increase in the intensity and a shifting toward lower wavenumbers of the peaks for amide I, II and III were noticed in the case of the samples with AuNPs, indicating a strong hydrogen bonding interaction between the chitosan and AuNPs [45,46,47,48,49]. Additionally, even though it was not as evident as for the amine group, a slight shift of the O-H in-plane bending was observed from 1419 cm^−1^ toward lower wavenumbers, which was due to the interaction of Au^0^ with the OH groups of chitosan [50,51]. The peak-shape similarity of pristine chitosan and the AuNP samples was an indicator of the uniform coating of the AuNPs with chitosan. The same remarks apply to Figure 4b for the FTIR spectra of the lyophilized samples with different gold concentrations and PG chitosan. The changes reflected the interaction degree of the AuNPs with PG and seemed to be proportional to the concentration of gold (Table 3).

**Figure 5:**
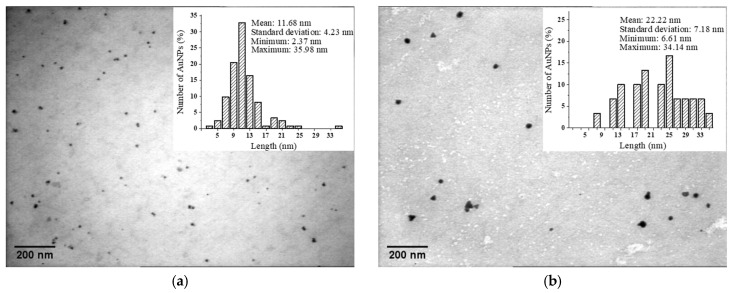

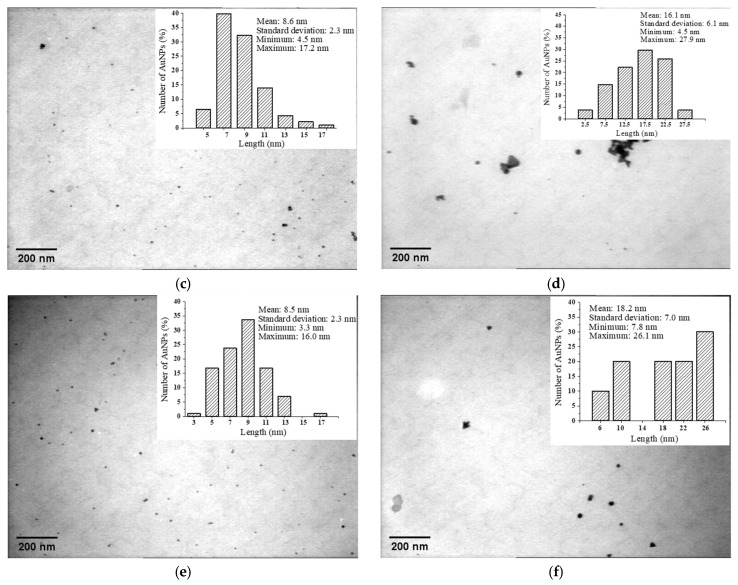
TEM image and size distribution for: (**a**) P 0.06; (**b**) P 0.12; (**c**) M 0.06; (**d**) M 0.12; (**e**) L 0.06; (**f**) L 0.12.

The size distribution analysis performed on the TEM micrographs (Figure 5) showed that the average size of the AuNPs was 11.7 nm for P 0.06, 8.6 nm for M 0.06 and 8.5 nm for L 0.06. With an increase in the gold concentration, we noticed larger nanoparticles (22.2 nm for P 0.12, 16.1 nm for M 0.12 and 18.2 nm for L 0.12) and the appearance of other geometric shapes (e.g., triangles).

**Figure 6:**
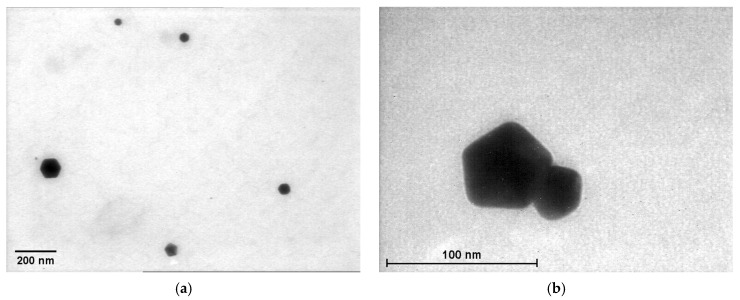

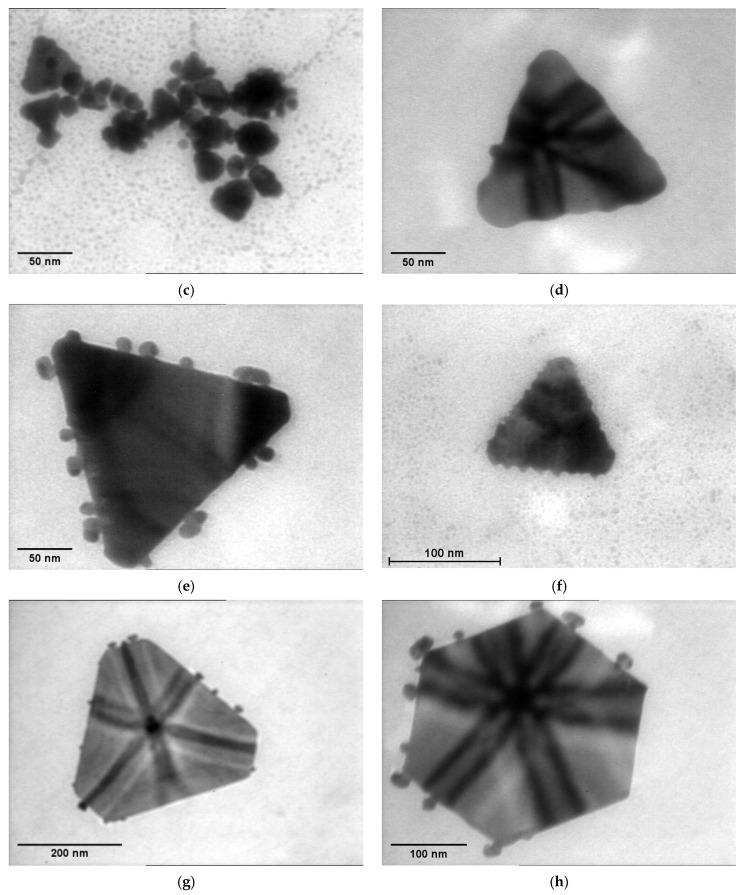
Examples of TEM images capturing the Ostwald ripening process for: (**a**) P 0.18; (**b**) P 0.24; (**c**) M 0.18; (**d**) M 0.24; (**e**) M 0.30; (**f**) L 0.18; (**g**) L 0.24; (**h**) L 0.30.

The TEM analysis showed a variety of geometric shapes obtained from the AuNPs of different samples, especially for those at a higher gold concentrations, such as spherical, hexagonal, tetrahedral and decahedral shapes (Figure 6). For the solutions containing gold concentrations of 0.24 or 0.30 mM, which precipitated, we captured images of Ostwald ripening that led to the growth of larger nanoparticles to the detriment of small ones, which dissolved. Thus, the nanoparticles reached a microscopic size (Figure 6). This process occurs when the surface of smaller particles is more energetically unstable than the surface of larger particles. As a consequence, they redeposit on the larger particles, which grow even larger. Over time, this causes an instable solution and phase separation.

The results regarding the AuNPs coated with the chitosan were confirmed by dynamic light scattering measurements (Table 4), which showed the average size of the nanoparticles formed in the chitosan aqueous solutions. For all the samples, an increase in the average hydrodynamic size of the AuNPs could be seen with an increase in the gold concentration after they were synthesized (month 0). In the case of the samples made using PG chitosan as a matrix, the average size remained consistent for a long period of time according to the measurements performed after 6 and 12 months.

When MMW or LMW chitosan were involved in obtaining the samples, it was noticed that the AuNP size varied on a greater scale after 6 months as the Ostwald ripening processes (such as the ones presented in the TEM images) still occurred in the solutions.

At gold concentrations of 0.24 and 0.30 mM, the solutions were not stable for more than 1 month, regardless of the type of chitosan used. As a consequence, the average size and zeta potential measurements could not be performed at 6 and 12 months.

An important physical parameter in characterizing the stability of colloidal solutions of AuNPs is the zeta potential [52], also measured by dynamic light scattering, which is influenced by the properties of the nanoparticle surface and the dispersion medium. The zeta potential value is given as a function of the electrostatic repulsion between charged nanoparticles. If the value is high (either positive or negative), it means that the repulsion forces between the nanoparticles are strong and this prevents their aggregation (the solution is stable). The data from Table 4 corresponding with the zeta potential of the solutions were all positive due to the amine groups of the chitosan. The zeta potential values obtained from the initial solutions were situated in the category of those with good stability for gold concentrations of 0.06, 0.12 and 0.18 mM (between 38 and 50 mV); the best results were obtained from the PG samples. For gold concentrations of 0.24 and 0.30 mM, the values were situated at the threshold of light dispersion or agglomeration.

After 6 months, the samples presented moderate or good stability and the size of the AuNPs was much more uniform. After 12 months, the stability of the solutions was moderate and tended to be situated at the threshold of light dispersion.

**Figure 7:**
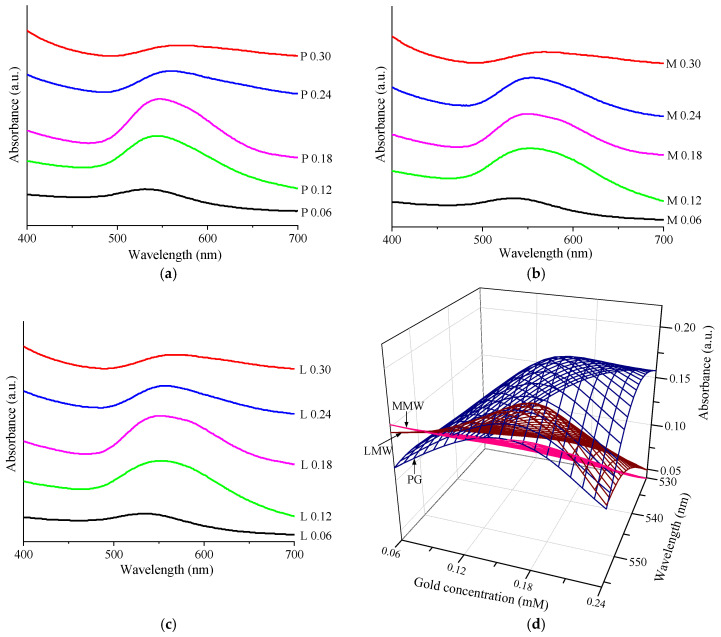
UV-Vis measurements: (**a**) UV-Vis spectra of PG samples; (**b**) UV-Vis spectra of MMW samples; (**c**) UV-Vis spectra of LMW samples; (**d**) the dependencies presented in Table 5.

Figure 7a–c show the UV-Vis spectra of the chitosan–AuNP colloidal samples for all the concentrations involved and different type of chitosan. It was found that these spectra had a SPR typical absorption band of AuNPs around 540 nm [25,53], which highlighted once again the presence of the AuNPs. Moreover, a shifting of this band maximum to longer wavelengths was observed as well as an increase in the absorption intensity with an increase in the precursor concentration and the chitosan molecular weight (Figure 7d); the values are summarized in Table 5. Figure 7d shows the dependencies presented in Table 5 between the gold concentration, the absorbance and the wavelength. For gold concentrations of 0.24 or 0.30 mM, the intensity decreased due to the drastic decrease in the Au^0^ concentration as a consequence of agglomeration and precipitation. In addition, at low gold concentrations, the maximum shifted to longer wavelengths as the mass of the chitosan was lower. The optimization of the process for AuNP synthesis could be realized considering these results.

**Figure 8:**
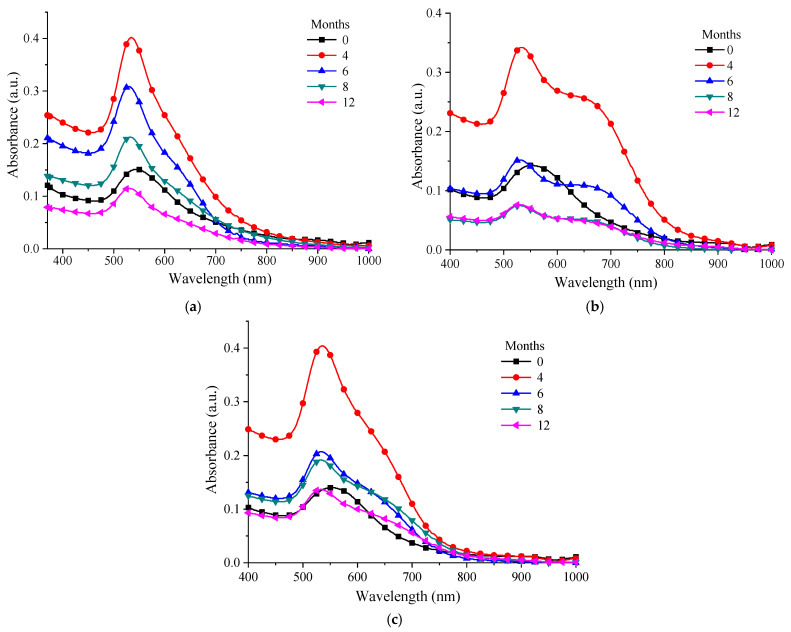
UV-Vis spectra over time for: (**a**) P 0.12; (**b**) M 0.12; (**c**) L 0.12.

In order to study the behavior in time of the chitosan–AuNP colloidal solutions, UV-Vis spectra were recorded after 4, 6, 8 and 12 months (Figure 8a–c). Thus, the spectra of the solutions recorded after 4 months confirmed that, in this period of time, the processes of maturation of the gold nanoparticles were still undergoing; the absorbance showed a significant increase in intensity compared with the spectrum recorded after the NP solution was prepared (month 0). After 6 months, it was observed that the absorbance began to decrease and the AuNPs had a tendency toward slight agglomeration and precipitation. Nevertheless, from the samples presented it could be seen that the best behavior was recorded for the P 0.12 sample, which was the sample obtained by using the chitosan with the highest molecular weight (PG). The other types of chitosan (MMW and LMW), although they presented an enhancement in the intensity of the plasmon resonance band after 4 months, exhibited a rapid decrease afterwards. This decrease in the absorbance value after 8–12 months was due to the drastic decrease in the AuNP concentration as a consequence of agglomeration and precipitation.

Furthermore, we investigated the SPR reactivity toward an increase in the refractive index of the surrounding environment of the AuNPs; this mechanism is involved in biosensing applications (biomolecular interaction sensing) that are based on colloidal gold nanomaterials [54,55,56]. Biosensors assume that an analyte is able to bind to a targeted receptor; its primary component is a physicochemical detector that will transduce the binding event into a measurable signal. Based on the detection mode, biosensors can include either a label (fluorophore or chromophore), a tracer (radioisotope) or a label-free analyte that reacts based on a physical parameter such as the refractive index, mass or thickness [55].

Considering that our AuNPs were created directly and through the aid of chitosan (meaning that this system could not be separated into its components), we had to find a way to enhance the photonic reactivity of the nanoparticles that were surrounded by the polymer. Thus, the AuNP solutions were mixed with glycerol solutions that led to a progressive increase in the refractive index of the medium from 1.33 to 1.41. The solutions were then investigated by UV-Vis spectroscopy to analyze if there was any possible modification in the absorbance or shifting of the peaks and by dark-field microscopy to visualize if the SPR of the AuNPs was enhanced after the refractive index was increased.

**Figure 9:**
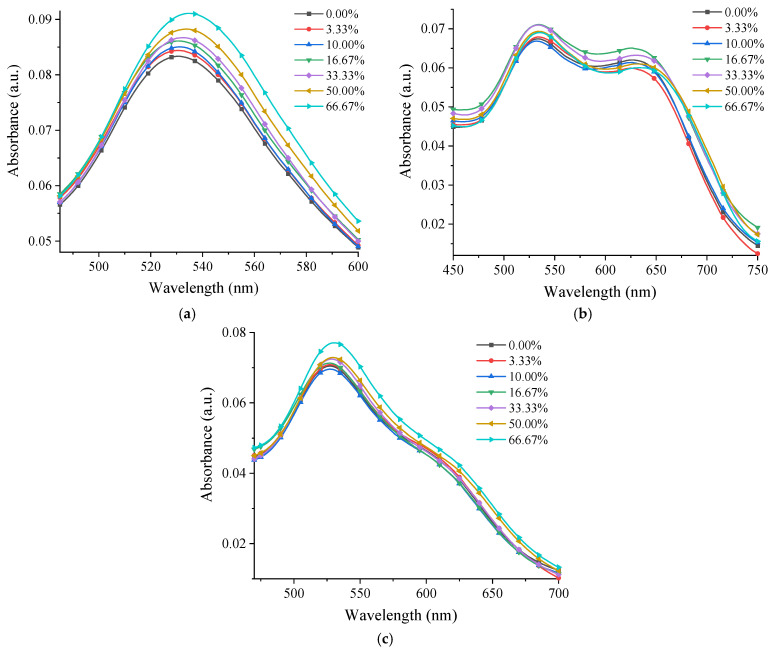
UV-Vis spectra at different glycerol concentrations for the samples: (**a**) P 0.12; (**b**) M 0.12; (**c**) L 0.12.

Figure 9 shows the UV-Vis spectra at different glycerol concentrations for the samples containing 0.12 mM gold and each type of chitosan. Although for all these samples an increase in the absorption intensity could be seen with an increase in the glycerol concentration, this took place progressively only for P 0.12. For M 0.12 and L 0.12, there was an alternation between the absorption intensity increasing and decreasing. The fact that the specific band of the AuNPs shifted to longer wavelengths in the case of the P 0.12 sample and the absorption intensity increased with an increase in the glycerol concentration demonstrated that the photonic reactivity of this composite system was enhanced [13,54].

**Figure 10:**
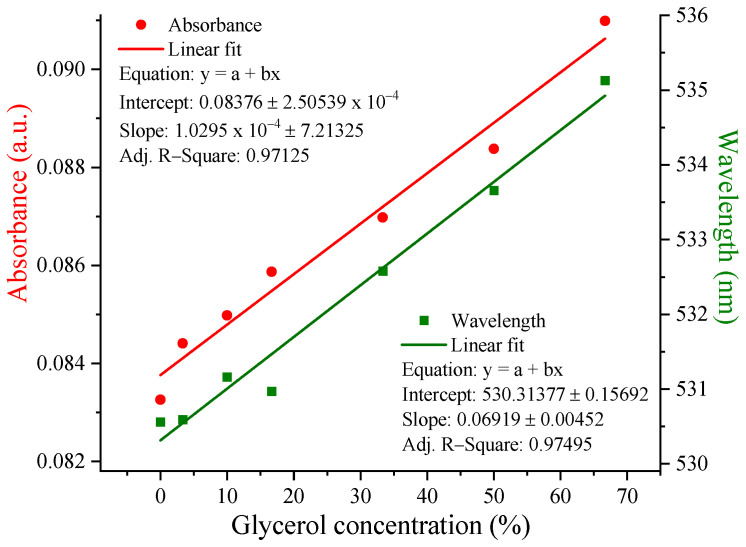
The UV-Vis maximum and wavelength dependence on glycerol concentrations for the P 0.12 sample.

When analyzing the spectra from Figure 9 and graphically representing the dependence of the UV-Vis maximum and wavelength on the different glycerol concentrations (Figure 10), it was observed that the band maximum shifted by ~6 nm for P 0.12 in a gradual manner, which did not happen for the other samples.

**Figure 11:**
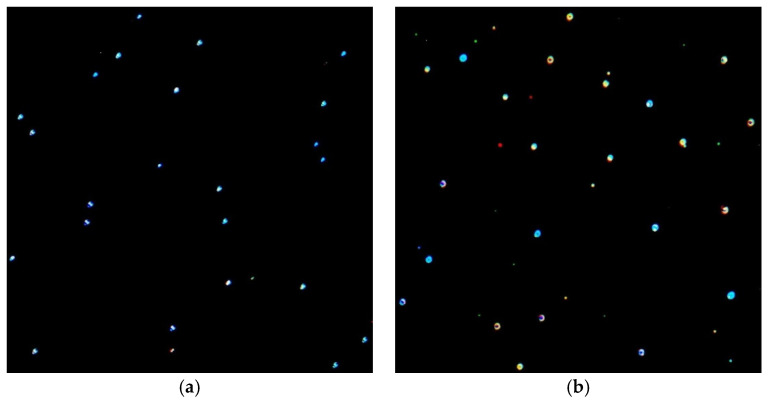
Micrographs obtained by dark-field optical microscopy that show the light scattered by AuNPs for P 0.12 sample: (**a**) without glycerol (0%) and (**b**) with a glycerol concentration of 50%. The magnification was 30×. Different-colored AuNPs highlight the fact that they had different geometries.

The differences in the shape or size distribution of the obtained AuNPs were also confirmed by dark-field optical microscopy micrographs (Figure 11). At a resonance, the SPR was maximum and the hollow size was at least one order of magnitude larger than the actual size of the nanoparticles, which made it possible for the AuNPs to be observed using dark-field optical microscopy. If noble metal nanoparticles are illuminated, the oscillation of the light electromagnetic field causes the oscillation of the metal-free electrons around the NP surface; this phenomenon leads to the separation of the charge and a dipole formation that oscillates parallel with the direction of the light electric field [50,57]. This oscillation has a maximum amplitude only at a certain frequency, named the surface plasmon resonance, which is stronger for plasmonic nanoparticles such as AuNPs. The resonance primarily depends on the local electric field at the NP surface and, thus, on the dielectric environment surrounding the NPs (chitosan nature and glycerol concentration) and their geometric form (the local electric field gradients that depend on the surface geometry). Therefore, NPs with different geometries will be differently colored, as described by the Mie theory [58]. As reported in the literature, AuNPs with a diameter in the range of 30–100 nm are easily visible under dark-field microscopy [59], the emission light being 5 orders higher than that of fluorescein molecules [60].

Figure 11a shows the dark-field microscopy image of the P 0.12 sample at a magnification of 30× in the absence of any glycerol amount; Figure 11b presents the image of P 0.12 for a glycerol concentration of 50%. As can be seen, there was a visible enhancement in the SPR of the AuNPs; these apparently had a larger size with more intense bright colors.

By the green and simple method that we developed, colloidal chitosan–gold nanoparticles that were stable in an aqueous solution for a long time (at least 12 months) were obtained. These composite nanoparticles were further improved in terms of photonic reactivity in respect to the refractive index increasing after adding known amounts of glycerol. The photonic performance of the new nanocomposite was enhanced in comparison with the initial AuNP system as a result of the electronic interaction of the materials in the final system. This new nanocomposite system offers new openings for biosensing applications because the increase in the SPR as a function of the surrounding medium refractive index is highly important in biomolecule interaction sensing using colloidal AuNPs [61,62].

The hypothesis in question was whether the new chitosan–AuNP–glycerol system could present an improved photonic reactivity, considering the polymer layers coating the AuNPs. This was verified and demonstrated by UV-Vis spectroscopy and dark-field optical microscopy. From the UV-Vis spectra, a progressive red shift of the maximum absorbance band by 6 nm and an increase in the absorbance intensity with an increase in the glycerol concentration were observed for the P 0.12 sample. Additionally, from the dark-field images, the photonic enhancement effect of the SPR was clearly evident.

## 4. Conclusions

We reported on the simple synthesis of nanocomposite colloidal solutions made of AuNPs encapsulated by different weights of chitosan. The nanoparticles were obtained by gold reduction and nucleation in the chitosan matrix after heating the solution under an ultrasonic field.

The main chemical bonds formed by the elements in the chitosan matrix were highlighted both by surface chemical analyses and Fourier transform infrared spectroscopy measurements.

The AuNPs had optical properties due to the SPR phenomenon, as evidenced by the UV-Vis analysis and dark-field microscopy. The method of synthesis presented in this paper led to stable gold–chitosan nanoparticles; the best results were obtained from the nanocomposite system made of PG chitosan with a gold concentration of 0.12 mM.

In conclusion, this proposed route of synthesis creates the possibility of producing gold–chitosan NPs that are stable in aqueous media and that can be stored for long periods.

Matching the properties of the individual synthesis materials used to obtain the nanocomposite allowed the charge transfers that occurred between them to subsequently absorb light in order to increase the photonic reactivity of the new composite system toward environmental changes in terms of the refractive index. The fact that the samples remained stable for a long time permits this application in biomedicine-related sciences.

## Figures and Tables

**Table 1:** The parameters of the samples.

Sample	Chitosan	Gold Concentration (mM)
P 0.06	PG	0.06
P 0.12	PG	0.12
P 0.18	PG	0.18
P 0.24	PG	0.24
P 0.30	PG	0.30
M 0.06	MMW	0.06
M 0.12	MMW	0.12
M 0.18	MMW	0.18
M 0.24	MMW	0.24
M 0.30	MMW	0.30
L 0.06	LMW	0.06
L 0.12	LMW	0.12
L 0.18	LMW	0.18
L 0.24	LMW	0.24
L 0.30	LMW	0.30

PG: practical grade; MMW: medium molecular weight; LMW: low molecular weight.

**Table 2:** The peaks of the characteristic FTIR bands from the fingerprint region of the chitosan samples and those with a gold concentration of 0.12 mM.

Sample	Wavenumber (cm^−1^)								
PG	1653	1557	1419	1376	1317	1154	1063	1026	892
P 0.12	1629	1523	1414	1378	1315	1152	1070	1027	894
MMW	1647	1567	1419	1375	1312	1150	1061	1025	893
M 0.12	1630	1555	1412	1378	1315	1151	1068	1031	895
LMW	1650	1568	1418	1374	1316	1150	1061	1025	893
L 0.12	1635	1546	1412	1381	1317	1152	1071	1032	894

**Table 3:** The peaks of the characteristic FTIR bands from the fingerprint region of PG and the samples with different gold concentrations.

Sample	Wavenumber (cm^−1^)								
PG	1653	1557	1419	1376	1317	1152	1063	1026	892
P 0.06	1634	1526	1416	1379	1316	1153	1065	1026	885
P 0.12	1629	1523	1414	1378	1315	1152	1070	1027	894
P 0.18	1625	1515	1413	1375	1314	1152	1062	1027	895
P 0.24	1619	1513	1412	1374	1313	1149	1061	1026	897
P 0.30	1617	1509	1404	1372	1311	1148	1060	1026	894

**Table 4:** The characterization of AuNPs in aqueous solutions: the average size and zeta potential of nanoparticles over time. The values are expressed as mean ± standard deviation.

Sample	Average Size (nm)			Average Zeta Potential (mV)		
	0 Month	6 Months	12 Months	0 Month	6 Months	12 Months
P 0.06	69.9 ± 5.7	70.2 ± 6.5	69.7 ± 6.2	47.2 ± 3.6	39.6 ± 3.4	36.4 ± 2.7
M 0.06	39.4 ± 3.1	76.2 ± 8.4	47.3 ± 5.9	42.4 ± 3.1	34.9 ± 2.9	35.6 ± 3.2
L 0.06	30.6 ± 4.7	83.3 ± 7.1	68.8 ± 6.3	40.4 ± 2.9	41.1 ± 3.7	35.2 ± 4.1
P 0.12	80.5 ± 9.2	77.9 ± 8.6	75.0 ± 8.9	49.7 ± 4.3	59.6 ± 4.8	28.8 ± 3.6
M 0.12	53.1 ± 5.4	63.7 ± 5.9	86.4 ± 7.7	41.5 ± 3.7	32.7 ± 3.1	28.9 ± 3.1
L 0.12	67.9 ± 6.1	76.1 ± 6.9	70.1 ± 7.3	37.8 ± 3.1	32.9 ± 3.9	31.5 ± 3.8
P 0.18	90.9 ± 7.8	80.6 ± 7.1	73.8 ± 6.9	41.2 ± 3.8	32.2 ± 2.8	26.3 ± 2.2
M 0.18	101.2 ± 9.4	82.5 ± 8.6	89.4 ± 9.3	39.8 ± 2.6	33.8 ± 3.6	13.1 ± 1.7
L 0.18	83.6 ± 6.8	102.0 ± 9.2	130.0 ± 14.9	38.8 ± 3.9	36.9 ± 3.2	23.4 ± 2.6
P 0.24	159.0 ± 22.4	-	-	29.9 ± 3.2	-	-
M 0.24	126.6 ± 14.8	-	-	24.8 ± 2.7	-	-
L 0.24	137.0 ± 23.1	-	-	20.3 ± 1.8	-	-
P 0.30	220.0 ± 29.9	-	-	5.11 ± 0.7	-	-
M 0.30	169.0 ± 20.7	-	-	14.8 ± 1.1	-	-
L 0.30	153.0 ± 17.6	-	-	11.3 ± 0.9	-	-

**Table 5:** The maximum plasmon resonance band values of the AuNPs as a function of gold concentration and type of chitosan.

Chitosan	Gold Concentration (mM)			
**Wavelength (nm)**	**0.06**	**0.12**	**0.18**	**0.24**
PG	530	543	548	556
MMW	536	553	554	552
LMW	536	552	553	555
